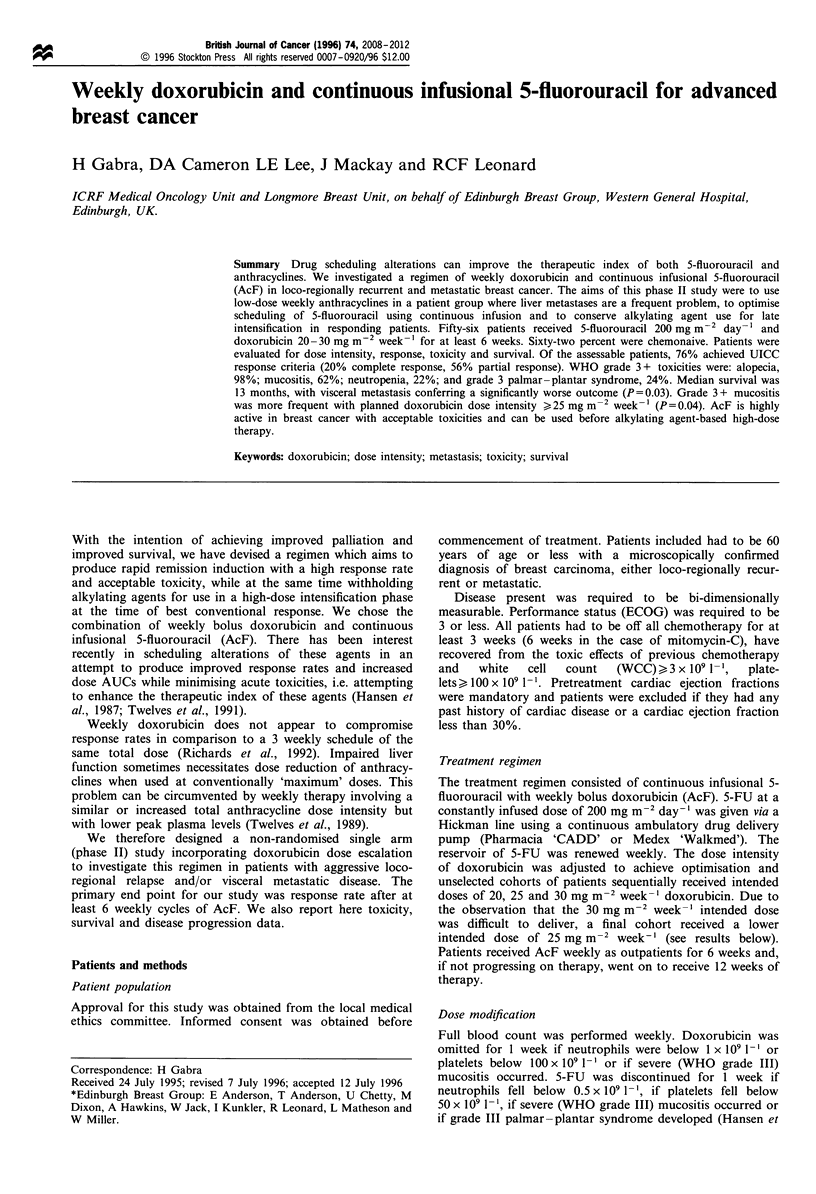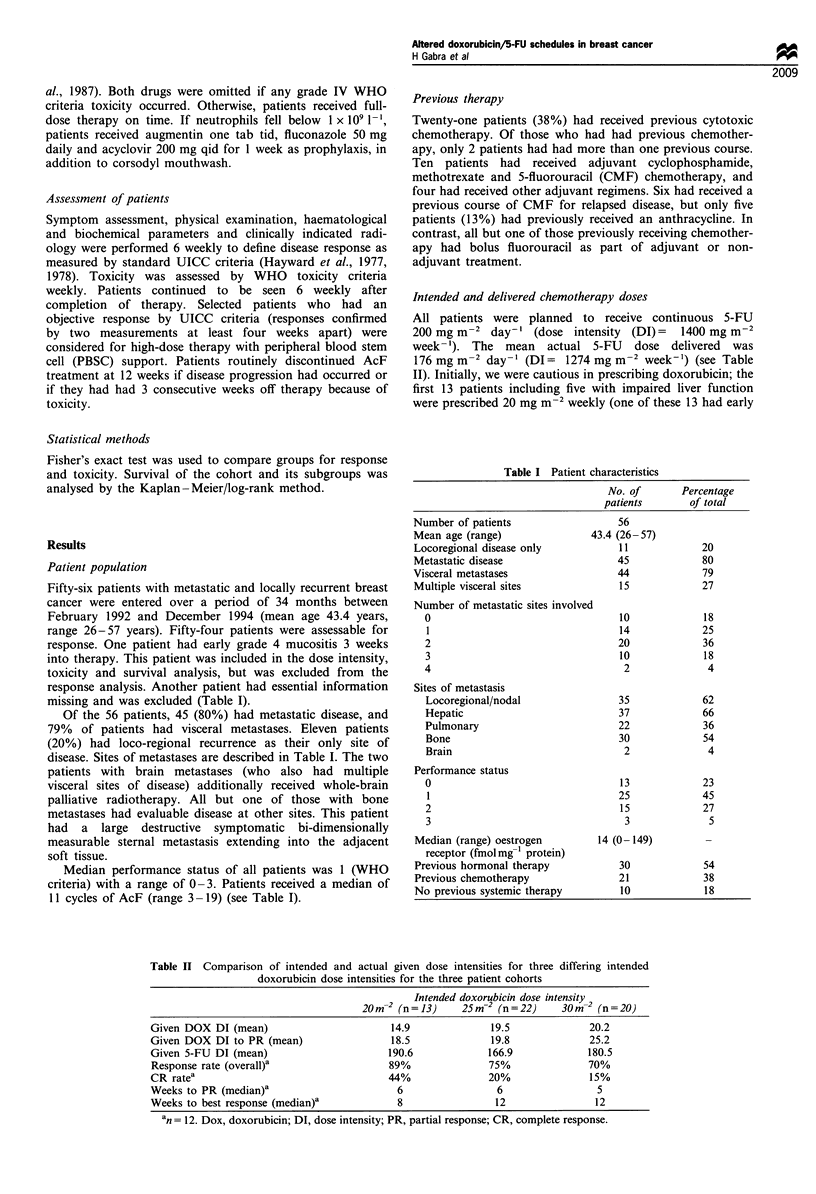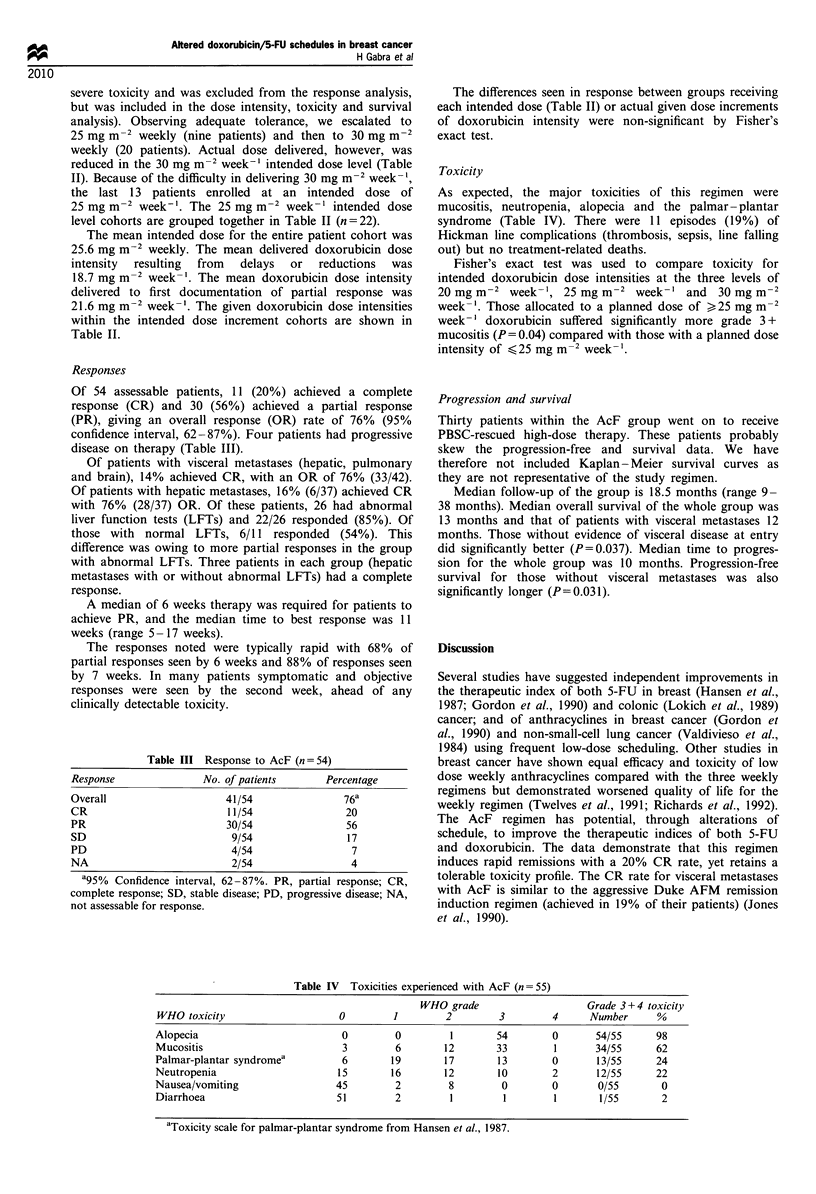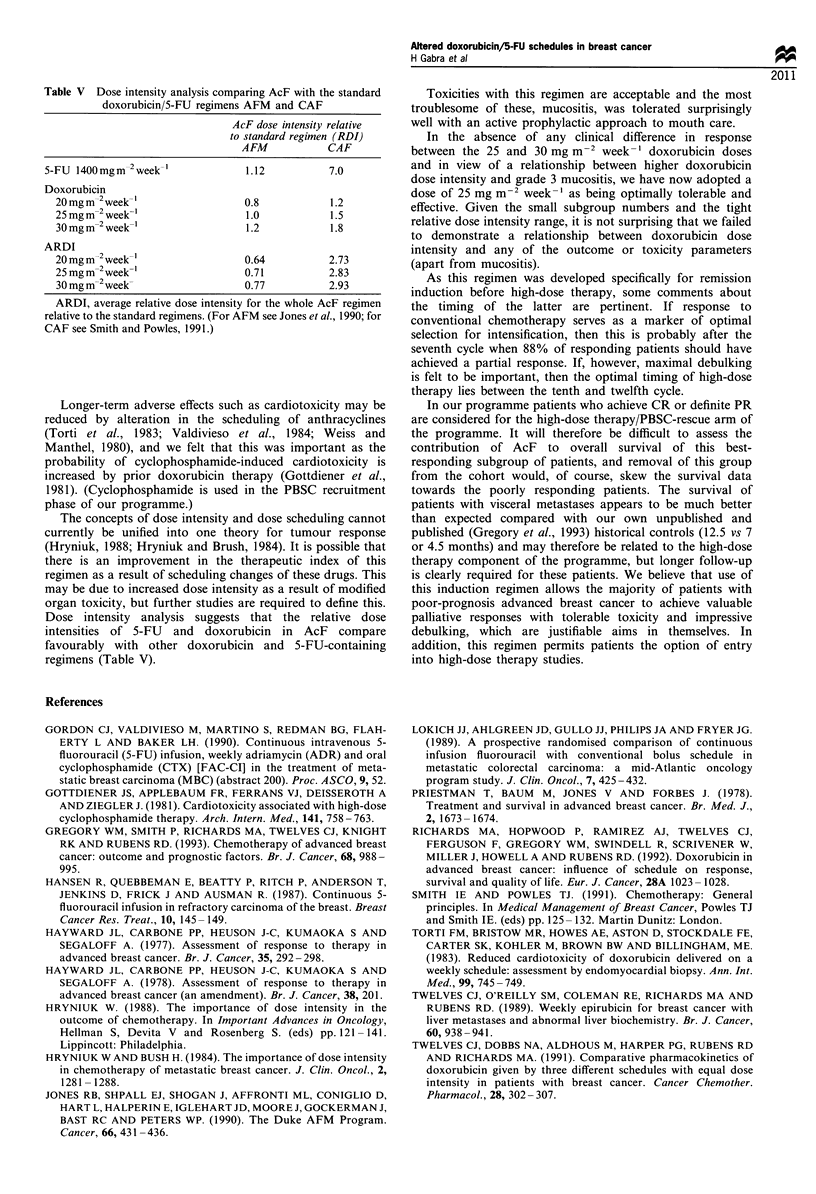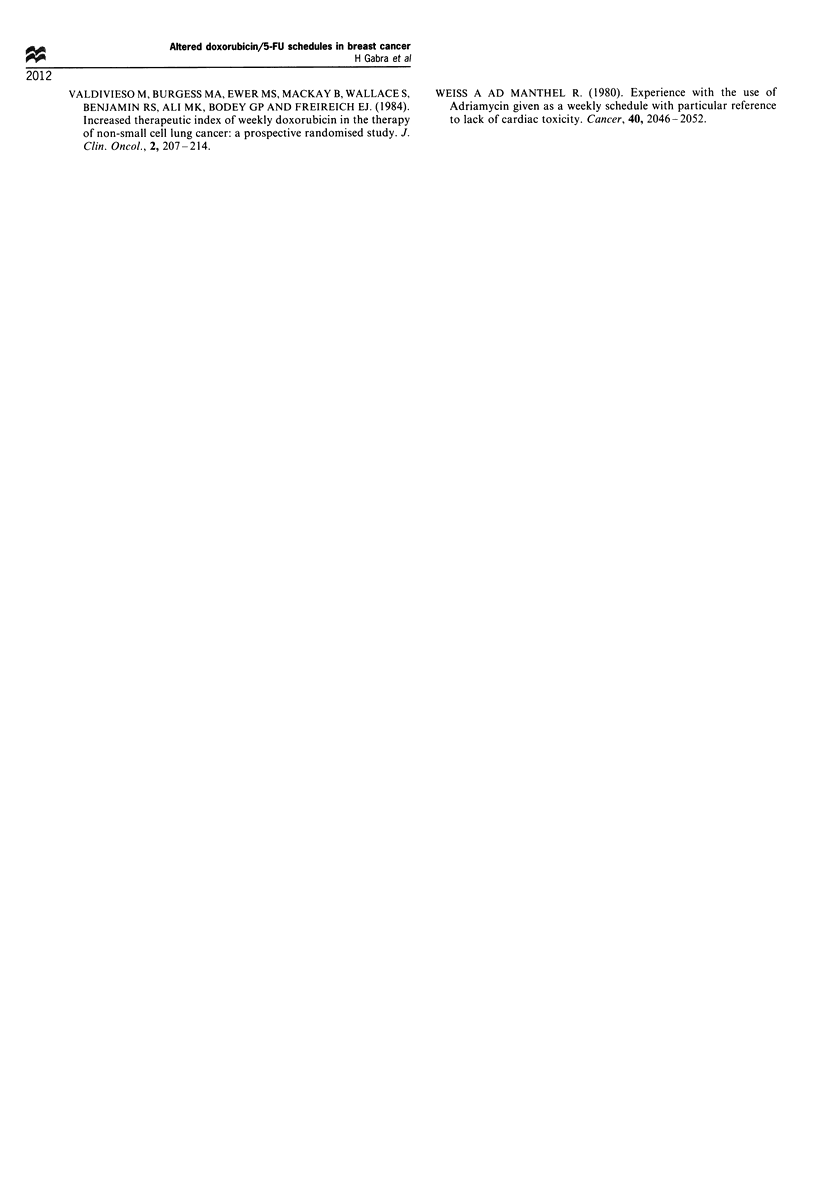# Weekly doxorubicin and continuous infusional 5-fluorouracil for advanced breast cancer.

**DOI:** 10.1038/bjc.1996.668

**Published:** 1996-12

**Authors:** H. Gabra, D. A. Cameron, L. E. Lee, J. Mackay, R. C. Leonard

**Affiliations:** ICRF Medical Oncology Unit, Western General Hospital, Edinburgh, UK.

## Abstract

Drug scheduling alterations can improve the therapeutic index of both 5-fluorouracil and anthracyclines. We investigated a regimen of weekly doxorubicin and continuous infusional 5-fluorouracil (AcF) in loco-regionally recurrent and metastatic breast cancer. The aims of this phase II study were to use low-dose weekly anthracyclines in a patient group where liver metastases are a frequent problem, to optimise scheduling of 5-fluorouracil using continuous infusion and to conserve alkylating agent use for late intensification in responding patients. Fifty-six patients received 5-fluorouracil 200 mg m-2 day-1 and doxorubicin 20-30 mg m-2 week-1 for at least 6 weeks. Sixty-two percent were chemonaive. Patients were evaluated for dose intensity, response, toxicity and survival. Of the assessable patients, 76% achieved UICC response criteria (20% complete response, 56% partial response). WHO grade 3+ toxicities were: alopecia, 98%; mucositis, 62%; neutropenia, 22%; and grade 3 palmar-plantar syndrome, 24%. Median survival was 13 months, with visceral metastasis conferring a significantly worse outcome (P = 0.03). Grade 3+ mucositis was more frequent with planned doxorubicin dose intensity > or = 25 mg m-2 week-1 (P = 0.04). AcF is highly active in breast cancer with acceptable toxicities and can be used before alkylating agent-based high-dose therapy.


British Journal of Cancer (1996) 74, 2008-2012
© 1996 Stockton Press All rights reserved 0007-0920/96 $12.00

### Weekly doxorubicin and continuous infusional 5-fluorouracil for advanced breast cancer

H Gabra, DA Cameron LE Lee, J Mackay and RCF Leonard

*ICRF Medical Oncology Unit and Longmore Breast Unit, on behalf of Edinburgh Breast Group, Western General Hospital, Edinburgh, UK.*

**Summary** Drug scheduling alterations can improve the therapeutic index of both 5-fluorouracil and anthracyclines. We investigated a regimen of weekly doxorubicin and continuous infusional 5-fluorouracil (AcF) in loco-regionally recurrent and metastatic breast cancer. The aims of this phase II study were to use low-dose weekly anthracyclines in a patient group where liver metastases are a frequent problem, to optimise scheduling of 5-fluorouracil using continuous infusion and to conserve alkylating agent use for late intensification in responding patients. Fifty-six patients received 5-fluorouracil 200 mg $m^{-2}$ $day^{-1}$ and doxorubicin 20-30 mg $m^{-2}$ $week^{-1}$ for at least 6 weeks. Sixty-two percent were chemonaive. Patients were evaluated for dose intensity, response, toxicity and survival. Of the assessable patients, 76% achieved UICC response criteria (20% complete response, 56% partial response). WHO grade 3+ toxicities were: alopecia, 98%; mucositis, 62%; neutropenia, 22%; and grade 3 palmar-plantar syndrome, 24%. Median survival was 13 months, with visceral metastasis conferring a significantly worse outcome ($P=0.03$). Grade 3+ mucositis was more frequent with planned doxorubicin dose intensity $\geqslant 25$ mg $m^{-2}$ $week^{-1}$ ($P=0.04$). AcF is highly active in breast cancer with acceptable toxicities and can be used before alkylating agent-based high-dose therapy.

**Keywords:** doxorubicin; dose intensity; metastasis; toxicity; survival

With the intention of achieving improved palliation and improved survival, we have devised a regimen which aims to produce rapid remission induction with a high response rate and acceptable toxicity, while at the same time withholding alkylating agents for use in a high-dose intensification phase at the time of best conventional response. We chose the combination of weekly bolus doxorubicin and continuous infusional 5-fluorouracil (AcF). There has been interest recently in scheduling alterations of these agents in an attempt to produce improved response rates and increased dose AUCs while minimising acute toxicities, i.e. attempting to enhance the therapeutic index of these agents (Hansen *et al.*, 1987; Twelves *et al.*, 1991).

Weekly doxorubicin does not appear to compromise response rates in comparison to a 3 weekly schedule of the same total dose (Richards *et al.*, 1992). Impaired liver function sometimes necessitates dose reduction of anthracyclines when used at conventionally 'maximum' doses. This problem can be circumvented by weekly therapy involving a similar or increased total anthracycline dose intensity but with lower peak plasma levels (Twelves *et al.*, 1989).

We therefore designed a non-randomised single arm (phase II) study incorporating doxorubicin dose escalation to investigate this regimen in patients with aggressive loco-regional relapse and/or visceral metastatic disease. The primary end point for our study was response rate after at least 6 weekly cycles of AcF. We also report here toxicity, survival and disease progression data.

### Patients and methods

### *Patient population*

Approval for this study was obtained from the local medical ethics committee. Informed consent was obtained before commencement of treatment. Patients included had to be 60 years of age or less with a microscopically confirmed diagnosis of breast carcinoma, either loco-regionally recurrent or metastatic.

Disease present was required to be bi-dimensionally measurable. Performance status (ECOG) was required to be 3 or less. All patients had to be off all chemotherapy for at least 3 weeks (6 weeks in the case of mitomycin-C), have recovered from the toxic effects of previous chemotherapy and white cell count (WCC)$\geqslant 3\times10^9$ $l^{-1}$, platelets$\geqslant 100\times10^9$ $l^{-1}$. Pretreatment cardiac ejection fractions were mandatory and patients were excluded if they had any past history of cardiac disease or a cardiac ejection fraction less than 30%.

### *Treatment regimen*

The treatment regimen consisted of continuous infusional 5-fluorouracil with weekly bolus doxorubicin (AcF). 5-FU at a constantly infused dose of 200 mg $m^{-2}$ $day^{-1}$ was given *via* a Hickman line using a continuous ambulatory drug delivery pump (Pharmacia 'CADD' or Medex 'Walkmed'). The reservoir of 5-FU was renewed weekly. The dose intensity of doxorubicin was adjusted to achieve optimisation and unselected cohorts of patients sequentially received intended doses of 20, 25 and 30 mg $m^{-2}$ $week^{-1}$ doxorubicin. Due to the observation that the 30 mg $m^{-2}$ $week^{-1}$ intended dose was difficult to deliver, a final cohort received a lower intended dose of 25 mg $m^{-2}$ $week^{-1}$ (see results below). Patients received AcF weekly as outpatients for 6 weeks and, if not progressing on therapy, went on to receive 12 weeks of therapy.

### *Dose modification*

Full blood count was performed weekly. Doxorubicin was omitted for 1 week if neutrophils were below $1\times10^9$ $l^{-1}$ or platelets below $100\times10^9$ $l^{-1}$ or if severe (WHO grade III) mucositis occurred. 5-FU was discontinued for 1 week if neutrophils fell below $0.5\times10^9$ $l^{-1}$, if platelets fell below $50\times10^9$ $l^{-1}$, if severe (WHO grade III) mucositis occurred or if grade III palmar-plantar syndrome developed (Hansen *et*

Correspondence: H Gabra
Received 24 July 1995; revised 7 July 1996; accepted 12 July 1996
*Edinburgh Breast Group: E Anderson, T Anderson, U Chetty, M Dixon, A Hawkins, W Jack, I Kunkler, R Leonard, L Matheson and W Miller.

al., 1987). Both drugs were omitted if any grade IV WHO criteria toxicity occurred. Otherwise, patients received full-dose therapy on time. If neutrophils fell below $1 \times 10^9 l^{-1}$, patients received augmentin one tab tid, fluconazole 50 mg daily and acyclovir 200 mg qid for 1 week as prophylaxis, in addition to corsodyl mouthwash.

*Assessment of patients*

Symptom assessment, physical examination, haematological and biochemical parameters and clinically indicated radiology were performed 6 weekly to define disease response as measured by standard UICC criteria (Hayward *et al.*, 1977, 1978). Toxicity was assessed by WHO toxicity criteria weekly. Patients continued to be seen 6 weekly after completion of therapy. Selected patients who had an objective response by UICC criteria (responses confirmed by two measurements at least four weeks apart) were considered for high-dose therapy with peripheral blood stem cell (PBSC) support. Patients routinely discontinued AcF treatment at 12 weeks if disease progression had occurred or if they had had 3 consecutive weeks off therapy because of toxicity.

*Statistical methods*

Fisher's exact test was used to compare groups for response and toxicity. Survival of the cohort and its subgroups was analysed by the Kaplan–Meier/log-rank method.

### Results

*Patient population*

Fifty-six patients with metastatic and locally recurrent breast cancer were entered over a period of 34 months between February 1992 and December 1994 (mean age 43.4 years, range 26–57 years). Fifty-four patients were assessable for response. One patient had early grade 4 mucositis 3 weeks into therapy. This patient was included in the dose intensity, toxicity and survival analysis, but was excluded from the response analysis. Another patient had essential information missing and was excluded (Table I).

Of the 56 patients, 45 (80%) had metastatic disease, and 79% of patients had visceral metastases. Eleven patients (20%) had loco-regional recurrence as their only site of disease. Sites of metastases are described in Table I. The two patients with brain metastases (who also had multiple visceral sites of disease) additionally received whole-brain palliative radiotherapy. All but one of those with bone metastases had evaluable disease at other sites. This patient had a large destructive symptomatic bi-dimensionally measurable sternal metastasis extending into the adjacent soft tissue.

Median performance status of all patients was 1 (WHO criteria) with a range of 0–3. Patients received a median of 11 cycles of AcF (range 3–19) (see Table I).

*Previous therapy*

Twenty-one patients (38%) had received previous cytotoxic chemotherapy. Of those who had had previous chemotherapy, only 2 patients had had more than one previous course. Ten patients had received adjuvant cyclophosphamide, methotrexate and 5-fluorouracil (CMF) chemotherapy, and four had received other adjuvant regimens. Six had received a previous course of CMF for relapsed disease, but only five patients (13%) had previously received an anthracycline. In contrast, all but one of those previously receiving chemotherapy had bolus fluorouracil as part of adjuvant or non-adjuvant treatment.

*Intended and delivered chemotherapy doses*

All patients were planned to receive continuous 5-FU 200 mg $m^{-2}$ $day^{-1}$ (dose intensity (DI) = 1400 mg $m^{-2}$ $week^{-1}$). The mean actual 5-FU dose delivered was 176 mg $m^{-2}$ $day^{-1}$ (DI = 1274 mg $m^{-2}$ $week^{-1}$) (see Table II). Initially, we were cautious in prescribing doxorubicin; the first 13 patients including five with impaired liver function were prescribed 20 mg $m^{-2}$ weekly (one of these 13 had early

**Table I** Patient characteristics

| | *No. of patients* | *Percentage of total* |
|---|---|---|
| Number of patients | 56 | |
| Mean age (range) | 43.4 (26–57) | |
| Locoregional disease only | 11 | 20 |
| Metastatic disease | 45 | 80 |
| Visceral metastases | 44 | 79 |
| Multiple visceral sites | 15 | 27 |
| Number of metastatic sites involved | | |
| 0 | 10 | 18 |
| 1 | 14 | 25 |
| 2 | 20 | 36 |
| 3 | 10 | 18 |
| 4 | 2 | 4 |
| Sites of metastasis | | |
| Locoregional/nodal | 35 | 62 |
| Hepatic | 37 | 66 |
| Pulmonary | 22 | 36 |
| Bone | 30 | 54 |
| Brain | 2 | 4 |
| Performance status | | |
| 0 | 13 | 23 |
| 1 | 25 | 45 |
| 2 | 15 | 27 |
| 3 | 3 | 5 |
| Median (range) oestrogen receptor (fmol $mg^{-1}$ protein) | 14 (0–149) | – |
| Previous hormonal therapy | 30 | 54 |
| Previous chemotherapy | 21 | 38 |
| No previous systemic therapy | 10 | 18 |

**Table II** Comparison of intended and actual given dose intensities for three differing intended doxorubicin dose intensities for the three patient cohorts

| | *Intended doxorubicin dose intensity* 20 $m^{-2}$ (n=13) | 25 $m^{-2}$ (n=22) | 30 $m^{-2}$ (n=20) |
|---|---|---|---|
| Given DOX DI (mean) | 14.9 | 19.5 | 20.2 |
| Given DOX DI to PR (mean) | 18.5 | 19.8 | 25.2 |
| Given 5-FU DI (mean) | 190.6 | 166.9 | 180.5 |
| Response rate (overall)[a] | 89% | 75% | 70% |
| CR rate[a] | 44% | 20% | 15% |
| Weeks to PR (median)[a] | 6 | 6 | 5 |
| Weeks to best response (median)[a] | 8 | 12 | 12 |

[a]$n$ = 12. Dox, doxorubicin; DI, dose intensity; PR, partial response; CR, complete response.

severe toxicity and was excluded from the response analysis, but was included in the dose intensity, toxicity and survival analysis). Observing adequate tolerance, we escalated to 25 mg m$^{-2}$ weekly (nine patients) and then to 30 mg m$^{-2}$ weekly (20 patients). Actual dose delivered, however, was reduced in the 30 mg m$^{-2}$ week$^{-1}$ intended dose level (Table II). Because of the difficulty in delivering 30 mg m$^{-2}$ week$^{-1}$, the last 13 patients enrolled at an intended dose of 25 mg m$^{-2}$ week$^{-1}$. The 25 mg m$^{-2}$ week$^{-1}$ intended dose level cohorts are grouped together in Table II ($n = 22$).

The mean intended dose for the entire patient cohort was 25.6 mg m$^{-2}$ weekly. The mean delivered doxorubicin dose intensity resulting from delays or reductions was 18.7 mg m$^{-2}$ week$^{-1}$. The mean doxorubicin dose intensity delivered to first documentation of partial response was 21.6 mg m$^{-2}$ week$^{-1}$. The given doxorubicin dose intensities within the intended dose increment cohorts are shown in Table II.

*Responses*

Of 54 assessable patients, 11 (20%) achieved a complete response (CR) and 30 (56%) achieved a partial response (PR), giving an overall response (OR) rate of 76% (95% confidence interval, 62–87%). Four patients had progressive disease on therapy (Table III).

Of patients with visceral metastases (hepatic, pulmonary and brain), 14% achieved CR, with an OR of 76% (33/42). Of patients with hepatic metastases, 16% (6/37) achieved CR with 76% (28/37) OR. Of these patients, 26 had abnormal liver function tests (LFTs) and 22/26 responded (85%). Of those with normal LFTs, 6/11 responded (54%). This difference was owing to more partial responses in the group with abnormal LFTs. Three patients in each group (hepatic metastases with or without abnormal LFTs) had a complete response.

A median of 6 weeks therapy was required for patients to achieve PR, and the median time to best response was 11 weeks (range 5–17 weeks).

The responses noted were typically rapid with 68% of partial responses seen by 6 weeks and 88% of responses seen by 7 weeks. In many patients symptomatic and objective responses were seen by the second week, ahead of any clinically detectable toxicity.

**Table III** Response to AcF ($n = 54$)

| *Response* | *No. of patients* | *Percentage* |
|---|---|---|
| Overall | 41/54 | 76[a] |
| CR | 11/54 | 20 |
| PR | 30/54 | 56 |
| SD | 9/54 | 17 |
| PD | 4/54 | 7 |
| NA | 2/54 | 4 |

[a]95% Confidence interval, 62–87%. PR, partial response; CR, complete response; SD, stable disease; PD, progressive disease; NA, not assessable for response.

The differences seen in response between groups receiving each intended dose (Table II) or actual given dose increments of doxorubicin intensity were non-significant by Fisher's exact test.

*Toxicity*

As expected, the major toxicities of this regimen were mucositis, neutropenia, alopecia and the palmar–plantar syndrome (Table IV). There were 11 episodes (19%) of Hickman line complications (thrombosis, sepsis, line falling out) but no treatment-related deaths.

Fisher's exact test was used to compare toxicity for intended doxorubicin dose intensities at the three levels of 20 mg m$^{-2}$ week$^{-1}$, 25 mg m$^{-2}$ week$^{-1}$ and 30 mg m$^{-2}$ week$^{-1}$. Those allocated to a planned dose of $\geqslant$25 mg m$^{-2}$ week$^{-1}$ doxorubicin suffered significantly more grade 3+ mucositis ($P = 0.04$) compared with those with a planned dose intensity of $\leqslant$25 mg m$^{-2}$ week$^{-1}$.

*Progression and survival*

Thirty patients within the AcF group went on to receive PBSC-rescued high-dose therapy. These patients probably skew the progression-free and survival data. We have therefore not included Kaplan–Meier survival curves as they are not representative of the study regimen.

Median follow-up of the group is 18.5 months (range 9–38 months). Median overall survival of the whole group was 13 months and that of patients with visceral metastases 12 months. Those without evidence of visceral disease at entry did significantly better ($P = 0.037$). Median time to progression for the whole group was 10 months. Progression-free survival for those without visceral metastases was also significantly longer ($P = 0.031$).

### Discussion

Several studies have suggested independent improvements in the therapeutic index of both 5-FU in breast (Hansen *et al.*, 1987; Gordon *et al.*, 1990) and colonic (Lokich *et al.*, 1989) cancer; and of anthracyclines in breast cancer (Gordon *et al.*, 1990) and non-small-cell lung cancer (Valdivieso *et al.*, 1984) using frequent low-dose scheduling. Other studies in breast cancer have shown equal efficacy and toxicity of low dose weekly anthracyclines compared with the three weekly regimens but demonstrated worsened quality of life for the weekly regimen (Twelves *et al.*, 1991; Richards *et al.*, 1992). The AcF regimen has potential, through alterations of schedule, to improve the therapeutic indices of both 5-FU and doxorubicin. The data demonstrate that this regimen induces rapid remissions with a 20% CR rate, yet retains a tolerable toxicity profile. The CR rate for visceral metastases with AcF is similar to the aggressive Duke AFM remission induction regimen (achieved in 19% of their patients) (Jones *et al.*, 1990).

**Table IV** Toxicities experienced with AcF ($n = 55$)

| | *WHO grade* | | | | | *Grade 3+4 toxicity* | |
|---|---|---|---|---|---|---|---|
| *WHO toxicity* | *0* | *1* | *2* | *3* | *4* | *Number* | *%* |
| Alopecia | 0 | 0 | 1 | 54 | 0 | 54/55 | 98 |
| Mucositis | 3 | 6 | 12 | 33 | 1 | 34/55 | 62 |
| Palmar-plantar syndrome[a] | 6 | 19 | 17 | 13 | 0 | 13/55 | 24 |
| Neutropenia | 15 | 16 | 12 | 10 | 2 | 12/55 | 22 |
| Nausea/vomiting | 45 | 2 | 8 | 0 | 0 | 0/55 | 0 |
| Diarrhoea | 51 | 2 | 1 | 1 | 1 | 1/55 | 2 |

[a]Toxicity scale for palmar-plantar syndrome from Hansen *et al.*, 1987.

**Table V** Dose intensity analysis comparing AcF with the standard doxorubicin/5-FU regimens AFM and CAF

| | *AcF dose intensity relative to standard regimen (RDI)* | |
|---|---|---|
| | *AFM* | *CAF* |
| 5-FU 1400 $mg\,m^{-2}\,week^{-1}$ | 1.12 | 7.0 |
| Doxorubicin | | |
| 20 $mg\,m^{-2}\,week^{-1}$ | 0.8 | 1.2 |
| 25 $mg\,m^{-2}\,week^{-1}$ | 1.0 | 1.5 |
| 30 $mg\,m^{-2}\,week^{-1}$ | 1.2 | 1.8 |
| ARDI | | |
| 20 $mg\,m^{-2}\,week^{-1}$ | 0.64 | 2.73 |
| 25 $mg\,m^{-2}\,week^{-1}$ | 0.71 | 2.83 |
| 30 $mg\,m^{-2}\,week^{-}$ | 0.77 | 2.93 |

ARDI, average relative dose intensity for the whole AcF regimen relative to the standard regimens. (For AFM see Jones *et al.*, 1990; for CAF see Smith and Powles, 1991.)

Longer-term adverse effects such as cardiotoxicity may be reduced by alteration in the scheduling of anthracyclines (Torti *et al.*, 1983; Valdivieso *et al.*, 1984; Weiss and Manthel, 1980), and we felt that this was important as the probability of cyclophosphamide-induced cardiotoxicity is increased by prior doxorubicin therapy (Gottdiener *et al.*, 1981). (Cyclophosphamide is used in the PBSC recruitment phase of our programme.)

The concepts of dose intensity and dose scheduling cannot currently be unified into one theory for tumour response (Hryniuk, 1988; Hryniuk and Brush, 1984). It is possible that there is an improvement in the therapeutic index of this regimen as a result of scheduling changes of these drugs. This may be due to increased dose intensity as a result of modified organ toxicity, but further studies are required to define this. Dose intensity analysis suggests that the relative dose intensities of 5-FU and doxorubicin in AcF compare favourably with other doxorubicin and 5-FU-containing regimens (Table V).

Toxicities with this regimen are acceptable and the most troublesome of these, mucositis, was tolerated surprisingly well with an active prophylactic approach to mouth care.

In the absence of any clinical difference in response between the 25 and 30 $mg\,m^{-2}\,week^{-1}$ doxorubicin doses and in view of a relationship between higher doxorubicin dose intensity and grade 3 mucositis, we have now adopted a dose of 25 $mg\,m^{-2}\,week^{-1}$ as being optimally tolerable and effective. Given the small subgroup numbers and the tight relative dose intensity range, it is not surprising that we failed to demonstrate a relationship between doxorubicin dose intensity and any of the outcome or toxicity parameters (apart from mucositis).

As this regimen was developed specifically for remission induction before high-dose therapy, some comments about the timing of the latter are pertinent. If response to conventional chemotherapy serves as a marker of optimal selection for intensification, then this is probably after the seventh cycle when 88% of responding patients should have achieved a partial response. If, however, maximal debulking is felt to be important, then the optimal timing of high-dose therapy lies between the tenth and twelfth cycle.

In our programme patients who achieve CR or definite PR are considered for the high-dose therapy/PBSC-rescue arm of the programme. It will therefore be difficult to assess the contribution of AcF to overall survival of this best-responding subgroup of patients, and removal of this group from the cohort would, of course, skew the survival data towards the poorly responding patients. The survival of patients with visceral metastases appears to be much better than expected compared with our own unpublished and published (Gregory *et al.*, 1993) historical controls (12.5 *vs* 7 or 4.5 months) and may therefore be related to the high-dose therapy component of the programme, but longer follow-up is clearly required for these patients. We believe that use of this induction regimen allows the majority of patients with poor-prognosis advanced breast cancer to achieve valuable palliative responses with tolerable toxicity and impressive debulking, which are justifiable aims in themselves. In addition, this regimen permits patients the option of entry into high-dose therapy studies.

### References

GORDON CJ, VALDIVIESO M, MARTINO S, REDMAN BG, FLAHERTY L AND BAKER LH. (1990). Continuous intravenous 5-fluorouracil (5-FU) infusion, weekly adriamycin (ADR) and oral cyclophosphamide (CTX) [FAC-CI] in the treatment of metastatic breast carcinoma (MBC) (abstract 200). *Proc. ASCO*, **9**, 52.

GOTTDIENER JS, APPLEBAUM FR, FERRANS VJ, DEISSEROTH A AND ZIEGLER J. (1981). Cardiotoxicity associated with high-dose cyclophosphamide therapy. *Arch. Intern. Med.*, **141**, 758–763.

GREGORY WM, SMITH P, RICHARDS MA, TWELVES CJ, KNIGHT RK AND RUBENS RD. (1993). Chemotherapy of advanced breast cancer: outcome and prognostic factors. *Br. J. Cancer*, **68**, 988–995.

HANSEN R, QUEBBEMAN E, BEATTY P, RITCH P, ANDERSON T, JENKINS D, FRICK J AND AUSMAN R. (1987). Continuous 5-fluorouracil infusion in refractory carcinoma of the breast. *Breast Cancer Res. Treat.*, **10**, 145–149.

HAYWARD JL, CARBONE PP, HEUSON J-C, KUMAOKA S AND SEGALOFF A. (1977). Assessment of response to therapy in advanced breast cancer. *Br. J. Cancer*, **35**, 292–298.

HAYWARD JL, CARBONE PP, HEUSON J-C, KUMAOKA S AND SEGALOFF A. (1978). Assessment of response to therapy in advanced breast cancer (an amendment). *Br. J. Cancer*, **38**, 201.

HRYNIUK W. (1988). The importance of dose intensity in the outcome of chemotherapy. In *Important Advances in Oncology*, Hellman S, Devita V and Rosenberg S. (eds) pp. 121–141. Lippincott: Philadelphia.

HRYNIUK W AND BUSH H. (1984). The importance of dose intensity in chemotherapy of metastatic breast cancer. *J. Clin. Oncol.*, **2**, 1281–1288.

JONES RB, SHPALL EJ, SHOGAN J, AFFRONTI ML, CONIGLIO D, HART L, HALPERIN E, IGLEHART JD, MOORE J, GOCKERMAN J, BAST RC AND PETERS WP. (1990). The Duke AFM Program. *Cancer*, **66**, 431–436.

LOKICH JJ, AHLGREEN JD, GULLO JJ, PHILIPS JA AND FRYER JG. (1989). A prospective randomised comparison of continuous infusion fluorouracil with conventional bolus schedule in metastatic colorectal carcinoma: a mid-Atlantic oncology program study. *J. Clin. Oncol.*, **7**, 425–432.

PRIESTMAN T, BAUM M, JONES V AND FORBES J. (1978). Treatment and survival in advanced breast cancer. *Br. Med. J.*, **2**, 1673–1674.

RICHARDS MA, HOPWOOD P, RAMIREZ AJ, TWELVES CJ, FERGUSON F, GREGORY WM, SWINDELL R, SCRIVENER W, MILLER J, HOWELL A AND RUBENS RD. (1992). Doxorubicin in advanced breast cancer: influence of schedule on response, survival and quality of life. *Eur. J. Cancer*, **28A** 1023–1028.

SMITH IE AND POWLES TJ. (1991). Chemotherapy: General principles. In *Medical Management of Breast Cancer*, Powles TJ and Smith IE. (eds) pp. 125–132. Martin Dunitz: London.

TORTI FM, BRISTOW MR, HOWES AE, ASTON D, STOCKDALE FE, CARTER SK, KOHLER M, BROWN BW AND BILLINGHAM, ME. (1983). Reduced cardiotoxicity of doxorubicin delivered on a weekly schedule: assessment by endomyocardial biopsy. *Ann. Int. Med.*, **99**, 745–749.

TWELVES CJ, O'REILLY SM, COLEMAN RE, RICHARDS MA AND RUBENS RD. (1989). Weekly epirubicin for breast cancer with liver metastases and abnormal liver biochemistry. *Br. J. Cancer*, **60**, 938–941.

TWELVES CJ, DOBBS NA, ALDHOUS M, HARPER PG, RUBENS RD AND RICHARDS MA. (1991). Comparative pharmacokinetics of doxorubicin given by three different schedules with equal dose intensity in patients with breast cancer. *Cancer Chemother. Pharmacol.*, **28**, 302–307.

VALDIVIESO M, BURGESS MA, EWER MS, MACKAY B, WALLACE S, BENJAMIN RS, ALI MK, BODEY GP AND FREIREICH EJ. (1984). Increased therapeutic index of weekly doxorubicin in the therapy of non-small cell lung cancer: a prospective randomised study. *J. Clin. Oncol.*, **2,** 207–214.

WEISS A AD MANTHEL R. (1980). Experience with the use of Adriamycin given as a weekly schedule with particular reference to lack of cardiac toxicity. *Cancer*, **40,** 2046–2052.